# Emergence of Lipid Droplets in the Mechanisms of Carcinogenesis and Therapeutic Responses

**DOI:** 10.3390/cancers15164100

**Published:** 2023-08-14

**Authors:** Dominique Delmas, Alexia K. Cotte, Jean-Louis Connat, François Hermetet, Florence Bouyer, Virginie Aires

**Affiliations:** 1 UFR of Heatlh Sciences, Université de Bourgogne, 21000 Dijon, France; alexia.cotte@parazapharma.com (A.K.C.); jean-louis.connat@u-bourgogne.fr (J.-L.C.); francois.hermetet@u-bourgogne.fr (F.H.); florence.bouyer@u-bourgogne.fr (F.B.); virginie.aires02@u-bourgogne.fr (V.A.); 2 INSERM Research Center U1231—Bioactive Molecules and Health Research Group, Cancer and Adaptive Immune Response Team, 21000 Dijon, France; 3 Centre de Lutte Contre le Cancer Georges François Leclerc, 21000 Dijon, France

**Keywords:** lipid droplets, cancers, chemoresistance, biomarkers, lipid metabolism

## Abstract

**Simple Summary:**

Cancer cells are characterized by an increased energy metabolism, to cope with a high rate of proliferation. Recently, alterations in lipid metabolism have been recognized to be involved in tumor progression. Lipid droplets represent dynamic entities implied in such a phenomenon. This review aims to point out the metabolic pathways that are presumed to be engaged in cancer cell survival and growth in order to define potential biomarkers of tumor progression and new therapeutic strategies in the treatment of cancer.

**Abstract:**

Cancer shares common risk factors with cardiovascular diseases such as dyslipidemia, obesity and inflammation. In both cases, dysregulations of lipid metabolism occur, and lipid vesicles emerge as important factors that can influence carcinogenesis. In this review, the role of different lipids known to be involved in cancer and its response to treatments is detailed. In particular, lipid droplets (LDs), initially described for their role in lipid storage, exert multiple functions, from the physiological prevention of LD coalescence and regulation of endoplasmic reticulum homeostasis to pathological involvement in tumor progression and aggressiveness. Analysis of LDs highlights the importance of phosphatidylcholine metabolism and the diversity of lipid synthesis enzymes. In many cancers, the phosphatidylcholine pathways are disrupted, modifying the expression of genes coding for metabolic enzymes. Tumor microenvironment conditions, such as hypoxia, different types of stress or inflammatory conditions, are also important determinants of LD behavior in cancer cells. Therefore, LDs represent therapeutic targets in cancer, and many lipid mediators have emerged as potential biomarkers for cancer onset, progression, and/or resistance.

## 1. Introduction

Despite promising therapeutic strategies against cancers and improvements in the prevention of these, the mortality rate continues to rise. These therapeutic failures are notably linked to the establishment of resistance mechanisms by the tumors to the treatments. Among these, it has emerged in recent years that one of the mechanisms by which malignant cells escape treatment is the reprogramming of their energy metabolism, in order to support their uncontrolled proliferation. More specifically, dysregulations of lipid metabolism have been highlighted, defined by an increased synthesis and absorption of lipids that can be degraded to produce energy or be stored in various dynamic intra- and extracellular organelles (i.e., lipid droplets (LDs), exosomes). In addition, it is now possible to establish lipid profiles that discriminate healthy tissue from cancerous tissue and localized tumors from more advanced tumors based on lipid content. Indeed, various studies have shown that increased tumor growth was associated with increased lipid content in tumors at earlier stages of diseases and that vesicles such as LDs and extracellular vesicles (EVs) (microvesicles (MVs) and exosomes) seem to play important roles in both tumor progression and chemoresistance to anticancer drugs. These organelles have attracted the attention of the scientific community since they were shown to be present in several types of cancer cells.

The present review concentrates on the current knowledge of LDs, including their biogenesis and their involvement in cancer processes. Lastly, these vesicles also play an important role in chemoresistance and could constitute potential targets to improve chemotherapeutic drugs.

## 2. Lipid Droplets: Structure and Composition

### 2.1. Structure and Composition

**Figure 1:**
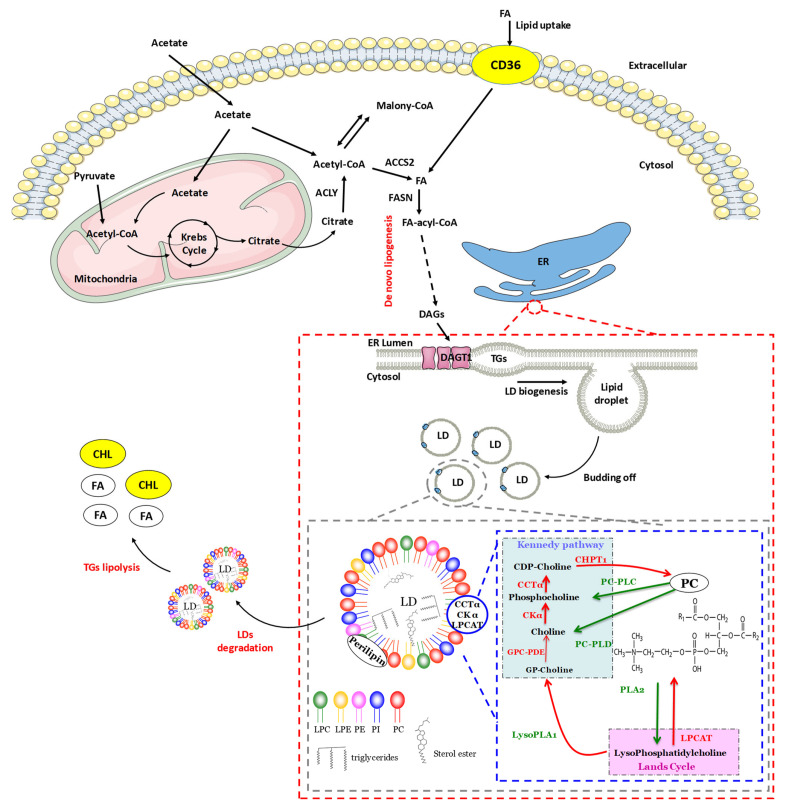
The formation of lipid droplets (LDs) results from the budding of the endoplasmic reticulum (ER) from the outer leaflet. Newly formed LDs can fuse together to increase their volume and surface area. These LDs are made up of very heterogeneous PLs where PC is predominant on the surface of LDs and thus prevents their coalescence, which is essential for the growth of LDs. Some of the enzymes of the Kennedy pathway and Land’s cycle seem to actively participate in the synthesis of PC at the level of LDs. In mammals, it can be synthesized de novo via two synthetic pathways [7] the main of which is the cytidine diphosphate-choline (CDP-choline) pathway, more commonly known as the Kennedy pathway. Choline is first phosphorylated to phosphocholine by choline kinase alpha (CKα) and then phosphocholine cytidylyltransferase alpha (CTTα), the limiting enzyme of this pathway, catalyzes the reaction between phosphocholine and cytidine triphosphate (CTP) to form CDP-choline which is then converted into PC by 1,2-diacylglycerol choline phosphotransferase (CHPT1) [8]. The second pathway for de novo synthesis of PC, known as the phosphatidylethanolamine N-methyltransferase (PEMT) pathway, occurs only in the liver and consists of a series of three PEMT-catalyzed PE methylations [9]. Finally, PC can also be synthesized via the remodeling of PLs during the Lands cycle by reacylation of LPC catalyzed by lysophosphatidylcholine acyltransferases 1, 2, and 4 (LPCAT1, LPCAT2 and LPCAT4) [10]. Ether from sterols is synthesized by coupling sterols with an FA using isoforms 1 and 2 of the enzyme acetyl-coenzyme A acetyltransferase (ACAT). The surface of LDs is composed of amphipathic and polar lipids forming a monolayer distinguishable from other cell membranes by their composition.

Lipid droplets (LDs) were originally described in cells that specialize in the storage of fatty acids (FAs) (e.g., adipocytes, hepatocytes, hormone-secreting cells), and the primary function of LDs is that of storing excess lipids. They are composed of a body of triacylglycerol (TAG) and a lesser amount of sterol esters (SEs). These neutral LDs are surrounded by a monolayer of phospholipids (PLs) and then packaged by a cage of the intermediate filament vimentin, a marker of mesenchymal origin cells. They are also widely described in pathological phenotypes such as hepatic steatosis and obesity [1]. The body of a TAG is synthesized from diacylglycerols (DAGs) using the two isoforms of the enzyme diacylglycerol acyltransferase 1 and 2 (DGAT1 and 2) [2]. The major PL in this monolayer is phosphatidylcholine (PC), followed by phosphatidylethanolamine (PE), and phosphatidylinositol (PI) (Figure 1). LDs are also enriched in lysophosphatidylcholine (LPC) and lysophosphatidylethanolamine (LPE) [3]. LDs present no or very limited quantities of phosphatidylserine (PS) and sphingolipids. We can also find non-esterified sterols and DAGs. LDs are also composed of a multitude of proteins participating or not in their formation and expansion. We can find structural proteins (e.g., proteins of the perilipin family), lipid synthesis enzymes (e.g., acetyl-CoA carboxylase ACC, DGAT2), lipases (e.g., adipose triglyceride lipase), and trafficking proteins (e.g., Rab5, Rab18) [4,5]. These proteins reside on the surface through different types of membrane interactions, but some can also transit between the surface and the body of neutral lipids such as perilipins [6].

### 2.2. Lipogenesis

#### 2.2.1. Initiation of Lipid Droplet Synthesis

Neutral lipids, found in the body of LDs, are synthesized at the level of the endoplasmic reticulum (ER). SEs and TAG derive from the successive action of enzymes of the family of membrane-bound-O-acyltransferases (MBOATs), including the ACAT and DGAT enzymes. These enzymes are mainly located in the ER and catalyze the condensation of endogenous or exogenous FAs with a DAG or a sterol. Certain isoforms such as DGAT2, 1-acylglycerol-3-phosphate O-acyltransferase 3 (AGPAT3) and AGPAT4 (Figure 1) can also be localized at the level of LDs, whereas GPAT1, AGPAT1, AGPAT2, and DGAT1 isoforms reside only at the ER [11]. Although the model for the accumulation of these lipids and the formation of LDs remains uncertain, several hypotheses have been put forward. The “canonical” model proposes that neutral lipids synthesized at the level of the ER accumulate within its membrane bilayer. A budding would form at this location, taking away the PLs from one of the sheets and resulting in the formation of new vesicles. The second hypothesis is also based on the accumulation of lipids between the two sheets of the membrane, but the budding would be replaced by an excision of the membrane [12]. The third possibility would be the formation of a budding of the ER bilayer. The vesicles formed would remain in contact with the ER to carry out neutral lipid exchanges in order to fill each vesicular space and form LDs (Figure 1) [13,14]. Other proteins have also been shown to be important, such as the protein spartin, encoded by the SPG20/SPART gene, which binds to ubiquitin ligases and is frequently associated with the LD surface acting to regulate the size and number [15]. SPG20 was suggested as a suitable biomarker for the detection of tumors at an early curable stage because it is hypermethylated in colorectal carcinomas (CRCs) and adenomas. Furthermore, spartin down regulation in cancer cells resulted in cytokinesis arrest [16]. More recently, Zhou et al. indicated that gastric cancer patients with poor spartin expression exhibited a worse prognostic than the high expression group [17]. Methylation of the gene induces SPG20 silencing and facilitates gastric cancer cell proliferation. A recent very good review details all the mechanisms by which spartin mediates its action [18].

Some studies have suggested that the enzymes responsible for the formation of neutral lipids may be located at the sites of the ER, forming sub-complexes [19]. However, it would seem that the DGAT1 and 2 isoforms are dispersed throughout the ER, suggesting an accumulation of neutral lipids in the form of spots [20]. Some studies have also shown that there are LDs preformation sites, as evidenced by the presence of various structural proteins.

How areas of neutral lipid accumulation are formed remains unclear, but the information gathered so far assumes that the simplest possible model would be that neutral lipids transit along the membrane sheets to reach the sites of preformed LDs. The accumulation of neutral lipids in LDs already formed and detached from the ER remains difficult to envisage because no investigations have found enzymes that would participate in the transfer of neutral lipids. The budding of LDs, meanwhile, may result from the biophysical properties of lipids. The composition as well as their size would lead to their instability, resulting in an emulsion in an aqueous phase which is the cytosol. This emulsion may be stabilized by the PLs present on the surface of the LDs, considered surfactants capable of reducing surface tension.

#### 2.2.2. Expansion of Lipid Droplets

Many enzymes participate in LD formation, heterogeneity, and lipolysis. Some proteins, containing a hydrophobic hairpin domain, have a dual localization between the ER and LDs. This is the case for proteins of the perilipin or seipin family [21,22]. Perilipin family proteins include: perilipin 1 (PLIN1), ADRP (also called adipophilin or PLIN2), TIP47 (tail-interacting protein of 47 kDa or PLIN3), S3-12 (or PLIN4) and OXPAT (or PPAR-induced LD protein (PAT) or PLIN5). These proteins can be overexpressed in certain cancers (e.g., lung cancer), actively participating in the formation of LDs in cancer cells [23]. In addition, overexpression of PLIN2 seems to be associated with increased incorporation of TAGs into LDs [24]. LDs can also grow thanks to a fusion phenomenon. In this case, several enzymes come into play: the proteins of the SNARE family (soluble N-ethylmaleimide-sensitive-factor attachment protein receptor), SNAP23 (synaptosomal-associated protein 23), syntaxin-5, and VAMP4 (vesicle-associated membrane protein 4) [25]. Newly formed LDs can continue to grow by coordinating their surface area and volume [26]. PC is the major PL on the surface of LDs and prevents their coalescence [27,28]. A study was able to show that the suppression of PC synthesis leads to a decrease in the surface/volume ratio, indicating that the quantity of PC produced is essential for the growth of LDs [29]. PC is synthesized via the Kennedy pathway and the Land’s cycle (Figure 1). The CDP-choline pathway plays a key role in cell cycle progression, cell proliferation and apoptosis [30]. In cancer, the PC synthesis pathways can be disrupted by modifying the expression of the genes coding for the involved enzymes, thus supporting oncogenesis [9]. Indeed, the overexpression of CKα, induced by certain oncogenic signaling pathways such as phosphatidylinositol 3-kinase (PI3K)/Ak strain transforming (Akt) [30], has been proven in many types of cancers, including CRC. In carcinoma of the larynx, overexpression of CCTα was positively correlated with the size and aggressiveness of the tumors, thus reducing the life expectancy of patients [31]. The same is true for the enzyme LPCAT1 in hepatocellular carcinoma [32] and breast cancer [33]. In addition, low expression of PEMT has been observed in liver and breast cancer cells, and is associated with rapid progression of these tumors [9].

Some of the enzymes in the Kennedy and Land’s pathways appear to actively participate in the synthesis of PC at the level of LDs. Specifically, CCTα preferentially binds to PC-depleted membranes which is why, during LD formation, their composition (which is similar to that of the ER) seems to stimulate the relocalization of the CCTα to the LDs [34]. In addition, it has been reported that the extinction of the CCTα enzyme in mammalian cells leads to an increase in the volume of LDs [35]. However, the CHPT1 enzyme, catalyzing the last stage of PC synthesis, is only located in the ER. So, the PC produced by this pathway would be mainly formed during LD budding. Some studies have also observed that LDs can establish contact with different organelles such as the ER [36]. This contact could make it possible to transfer proteins. The Lands cycle converts LPC to PC. One study showed that isoforms 1 and 2 were partly localized in LDs, participating directly in the synthesis and remodeling of PC in squamous cell carcinoma and liver cancer cells [37]. The remodeling of the different PLs found in LDs can also be modulated by the presence of different lipases that are specific to PLs, such as phospholipase A2 (PLA2) or D (PLD) [38,39].

### 2.3. Lipolysis

Lipolysis of LDs is controlled by adipose triglyceride lipase, hormone-sensitive lipase, and monoacylglycerol lipase enzymes, which hydrolyze TAGs, DAGs, and monoacylglycerols (MAGs), respectively, releasing a fatty acid each time [40]. The presence of perilipins, proteins of the CIDE family (cell death-inducing DNA fragmentation factor-45-like effector), or Fsp27 (fat-specific protein 27) dictates the accessibility of lipids to lipases. For example, in the context of lipid homeostasis, PLIN1 protects LDs from the action of lipases [41]. LD lipolysis can also be mediated by a form of autophagy called lipophagy [42,43]. The LDs are then encapsulated by the lysosomal vesicles where hydrolytic enzymes and proteases degrade the TAGs and the proteins present in the LDs.

### 2.4. Lipid Droplet Isolation and Quantification Methods

The gold standard method for LD isolation from multiple species and from cell culture or tissues, is based on differential centrifugation using discontinuous sucrose gradients [44]. Owing to their low protein and to their high lipid content, LDs are isolated as a low-density floating fat ring on all aqueous gradients after centrifugation. As a primary critical step, fresh cells or tissues after washings, must be disrupted in sucrose-containing buffers (generally 250 mM sucrose) using appropriate material (i.e., needles of adapted gauge according to cell size or dounce homogenizers for tissues), and most buffers contain inhibitors to preserve LDs from damage according to the type of subsequent analysis (i.e., proteases inhibitors for protein analysis). The limitation at this stage is that when using dounce homogenizers with fatty samples such as adipose tissue, the large LDs might be lost under the effect of the mechanically applied high pressure. A first centrifugation step from 1000 to 3000× *g* for up to 10 min at 4 °C is used to discard nuclei, cell debris, and unbroken material. The resulting supernatant containing LDs, which can be referred as the post-nuclear supernatant (PNS) fraction, is further used for ultracentrifugation at speeds (28,000 to 270,000× *g*) and at times (1–2 h) that can vary according to LDs size in samples [44,45]. This allows the separation of LDs from the other cellular compartments and the collection of the LD fraction from the top gradient. Thereafter, washing steps are required to ensure LD fraction purity and to separate cytosolic and total membrane fractions. To ensure isolation efficiency and purity, LDs can further be delipidated with acetone or chloroform/methanol solvents to precipitate proteins for subsequent Western blot analysis. Usually, equal proteins amounts are loaded on SDS-PAGE and antibodies directed to LDs markers such as perilipins or to total membrane and cytosolic fraction markers (i.e., caveolin-1 and Na^+^/K^+^ ATPase, respectively) are used. Additionally, confocal microscopy using fluorescent dyes such as Nile red or Bodipy^TM^ 493/503 (Thermo Fisher Scientific, Waltham, MA, USA), which display a high affinity to neutral lipids, can also be performed to control LD fraction purity and to assess LD size. In general, the size and abundance of LDs are determined by combining fluorescent confocal microscopy using the above-mentioned probes or by flow cytometry (Bodipy^TM^ 493/503 probe only) and transmission electron microscopy (TEM) approaches. Depending on the experimental readouts and sometimes on the nature of the biological sample, some modifications to the gold standard isolation protocol can be found, notably when assessing the LD proteome and lipidome by mass spectrometry techniques [45,46,47]. Currently, commercial LD isolation kits are also available, based on simple gradient centrifugation instead of ultracentrifugation, and using reduced sample sizes [48,49]. Other techniques, like coherent Raman scattering (CRS), are suggested as essential tools for profiling and quantifying intracellular LDs in a non-destructive and time-resolved fashion [50].

## 3. Lipid Droplets and Cancers

Many studies have focused on evaluating the function or functions of these LDs in carcinogenesis. Initially, some studies highlighted the involvement of LDs in the survival of cells under conditions of nutrient stress. When cancer cells have easy access to nutrients, they are able to take up FAs. Adipocytes are the primary storage areas for triglycerides and are already recognized for their ability to induce tumor growth and metastases, as demonstrated by a study showing that metastatic ovarian cancer cells were able to migrate to the adipocyte tissue of the omentum. This phenomenon was explained by the fact that the hydrolysis of adipocyte TAGs produces free FAs that are captured and reused as an energy source by ovarian cancer cells [51]. The transfer of FAs and cholesterol within the stroma is performed through lipoproteins, serum albumin and exosomes. FAs and cholesterol can be esterified in order to be integrated within LDs in anticipation of more restrictive conditions. These FAs can then be reused by β-oxidation, when the cancer cells see their energy needs increased and/or when the environmental conditions so require. In particular, hypoxia is a factor that induces the remobilization of FAs towards β-oxidation by activation of the CPT1 enzyme. Some studies have shown that the expression of CPT1 is elevated in hypoxic conditions and is associated with a reduction in the quantity of LDs [52]. In addition, LDs can create close contact with mitochondria in order to transfer FAs directly towards mitochondrial β-oxidation [53]. LDs may, among other things, be detected in breast cancer cells by a Raman spectroscopy method as a result of their accumulation [54].

### 3.1. Lipotoxicity Reduction/Induction Balance

Some studies have highlighted that down regulation of LDs or their protein content could reduce lipotoxicity in different pathological models. Indeed, a silencing of STE20-type kinase TAOK1, a hepatocellular LD-associated protein, reduces lipotoxicity in human hepatocytes and hepatocarcinoma cells by accelerating lipid catabolism (mitochondrial β-oxidation and TAG secretion), suppressing lipid anabolism (FA influx and lipogenesis), and mitigating oxidative/endoplasmic reticulum stress and modulating metabolic and pro-oncogenic signaling [55]. In this way, AKR1C3 is required to mitigate cellular lipotoxicity by promoting LD formation in a lipophagy-dependent manner in hepatocellular carcinoma tumors [56].

In the same manner, a reduction in LD aggregation prevents senescence in hypothalamic neural stem cells and could improve hypothalamic dysfunction in adamantinomatous craniopharyngioma (ACP) patients [57].

Very interestingly, in glioblastoma mouse models, targeting de novo lipid synthesis induces lipotoxicity and impairs DNA damage repair [58]. Other studies have shown that higher metabolic activity and excessive lipid accumulation are responsible for metabolic stress and lipotoxicity, inducing excessive accumulation of intracellular LDs through a proprotein convertase subtilisin/kexin type 9 (PCSK9) deficiency in hepatoma cells [59]. This deficiency of its enzyme induces TAG accumulation in LDs and subsequently leads to cell death. In glioblastoma multiforme (GBM), inhibition of TAG synthesis depletes LDs and increases lipotoxicity and reduced cell proliferation [60,61].

### 3.2. LDs and Renal Cancer

Numerous studies using renal carcinoma models have shown that cancer cells in normoxic conditions contain high quantities of LDs, which are associated with a poor prognosis [62]. Glutathione peroxidase 8 (GPX8), knockout reduces LD levels (independent of lipid uptake), FA de novo synthesis, and triglyceride esterification in clear cell renal cell carcinoma (ccRCC), thus constituting a potential target in this cancer model [63]. In the same manner, RNASET2 knockout inhibits DGAT1 and 2 and reduces LD accumulation, concomitant with suppression of cell proliferation, migration, and invasion in ccRCC [64]. Thus, pharmacological inhibition of DGAT1 could be a potential target in ccRCC [64,65]. In fact, various proteins could be targeted to disturb LD formation or accumulation for example, inhibition of the enzyme catalyzing esterification of FAs with coenzyme A, acyl-CoA synthetase 3 (ACSL3), disrupts LD accumulation in ccRCC and leads ferroptosis [66]. Inhibition of the elongation of very long-chain fatty acid 5 (ELOVL5) enzyme, which catalyzes polyunsaturated FA elongation, suppressed the formation of LDs and induced apoptosis via ER stress [67].

**Figure 2:**
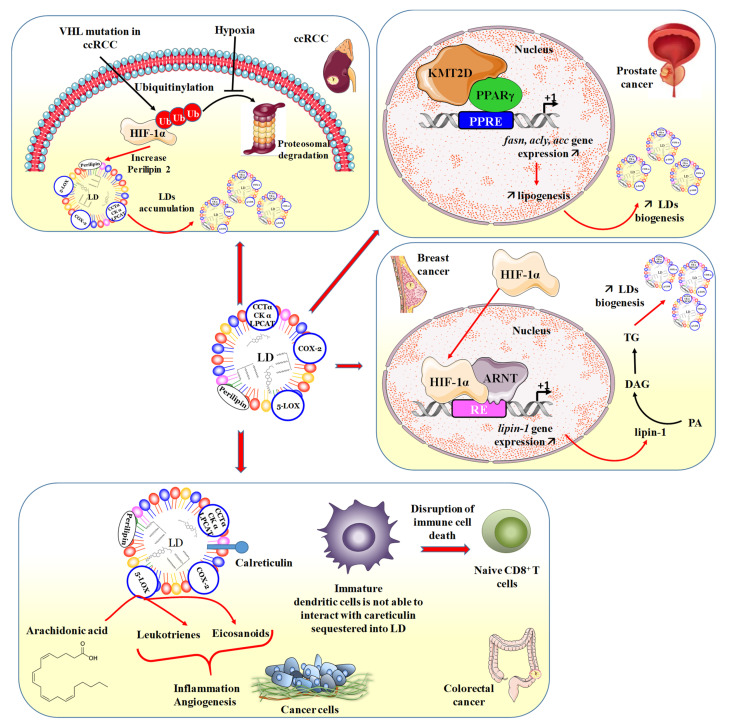
Involvement of LDs in different types of cancers where the accumulation of LDs is associated with the aggressiveness of cancers. In renal cancers, the VHL mutation favors the ubiquitination of HIF, thus allowing the increase in the expression of perilipin 2, which plays an essential role in the biogenesis of LDs. Moreover, the phenomenon of hypoxia comes to counter the proteosomal degradation of ubiquitylated HIF, thus reinforcing the action of the latter on the quantity of perilipin 2. Consequently, this results in the accumulation of LDs. In prostate cancer, the interaction of PPARγ with KMT2D promotes the activation of gene transcription such as *fasn*, *acly*, and *acc*, which makes it possible to increase lipogenesis and in fact the biogenesis of LDs that will accumulate. In the same way in breast cancer, HIF-1α activates the transcription of the *lipin-1* gene coding for the lipin-1 protein, whose enzymatic activity catalyzes the conversion of phosphatidic acid (PA) to DAG and consequently to LD biogenesis and its accumulation. In colon cancer, various mechanisms are involved through the enzymes present in LDs, such as COX-2 and LOX, which participate in inflammatory processes and tumor angiogenesis. Furthermore, the sequestration of calreticulin into LDs induces a failure in dendritic cell maturation, which leads to limited recruitment and activation of naïve CD8^+^ T cells and leads to a disruption of immune cell death.

The renal carcinoma model has the advantage, in humans, of being well vascularized. However, cancer cells exhibit constitutive activation of hypoxia inducible factors (HIFs), which is associated with LD production. LD accumulation appears to be caused by the intake of cholesterol as a result of the overexpression of the very low-density lipoproteins (VLDL) receptor generated by HIF1α [68]. HIF1α also targets HIG2 (hypoxia-inducible protein 2) and PLIN2, which are two proteins associated with LD formation. In fact, the low bioavailability of oxygen within a solid tumor leads to the activation of HIFs. Sometimes, mutations in the von Hippel Lindau (VHL) protein led to the constitutive activation of HIF1α and HIF2α (e.g., renal carcinomas) (Figure 2) [69]. The most common effect of HIF is the induction of lipid biogenesis (Figure 2). Hypoxia-dependent HIF-2α also promotes the remodeling of lipid metabolism and the malignant phenotype of ccRCC via CD36 [70]. Furthermore, HIF2α-dependent PLIN2 expression promoted lipid storage, proliferation, and viability in xenograft ccRCC tumors, and this mechanism involved an ER stress [71].

Hypoxia is also responsible for increasing the uptake of lipids containing monounsaturated FAs [72]. It promotes the expression of proteins FABP3 and 7 (FA binding proteins), which are involved in the transport and trafficking of FAs. HIF1 can promote neutral lipid storage in LDs through activation of HIG2 [73]. HIF1α also induces the storage of FAs in LDs through the activation of PLIN2 [74]. Thus, the induction of PLIN2 by HIF2α is responsible for the production of LDs and the survival of so-called ccRCC cells. Survival mediated by the HIF2α-PLIN2 axis would be due to the maintenance of ER homeostasis (Figure 2) [71]. During the induction of ER stress, the protein elimination mechanism called ERAD (endoplasmic-reticulum-associated protein degradation) can compensate for the accumulation of misfolded proteins. Some studies have underlined the numerous interactions between LDs and other organelles. It would seem that LD-associated proteins (e.g., CIDE) can be associated with both the ER and the proteasome, suggesting their involvement in maintaining ER homeostasis by facilitating ERAD [75]. Environmental fluctuations lead to the mobilization, uptake, or transfer of Fas, which can give rise to a lipotoxic phenomenon. However, it seems that not all cells within the same population of cancer cells are able to prevent lipotoxicity. This ability is associated with the production of LDs. Indeed, cells accumulating LDs are also able to accumulate reactive oxygen species (ROS) and reduce lipotoxicity within their population (Figure 2) [76].

### 3.3. LDs and Prostate Cancer

In a model of prostate cancer, the formation of LDs appears to be regulated by an autophagy mechanism. Specifically, during androgen deprivation, LDs are degraded by the mechanism of lipophagy and cell growth is maintained under these conditions [77].

In this type of cancer, the use of alpinumisoflavone, could disrupt intracellular FAs, cholesterols, and LD contents in prostate cancer cells through an inhibition of FASN and 3-hydroxy-3-methylglutaryl-CoA reductase (HMGCR) [78]. Prostate cancer, like breast cancer, is strongly subject to hormonal status, so recent studies have been able to show that androgens deregulate lipid metabolism and enhance the effects of LDL increasing prostate cancer cell viability in which LDL and 5α-dihydrotestosterone synergistically enhanced the accumulation of LDs [79]. The use of androgen antagonists such as proxalutamide is able to alter SREBP-1/FASN/lipogenesis and androgen signal axis and subsequently decrease LD biogenesis and constitutes a promising approach to decrease prostate cancer cell proliferation and invasion [80] Very interestingly, transcriptional factor peroxisome proliferator-activated receptor gamma (PPARγ) was able to interact with kinase tether histone-lysine N-methyltransferase 2D (KMT2D) to affect lipid synthesis and subsequently LDs (Figure 2) [81]. These observations could constitute a potential therapeutic target through the use of inhibitors of PPARγ and KMT2D.

### 3.4. LDs and Breast Cancer

The production of LDs is also observed in some breast cancers and is, associated with the presence of hormone receptors in cancer cells. This production appears to be induced during hormone-based treatment (e.g., progestin) [82]. However, triple-negative breast cancer cells may also exhibit LDs production associated with increased proliferation and motility. In this case, production appears to be induced by stimulation via saturated Fas, leading to the activation of the PLA2 enzyme or by peroxisome proliferator-activated receptor (PPARγ) ligands [83,84].

In particular, HIF1 can induce the expression of the fatty acid synthase gene (FASN) in breast cancer lines [85] or of acyl-CoA synthetase 2 (ACS2), synthesizing the formation of acetyl-CoA from cytoplasmic acetate [86]. HIF1α is also capable of indirectly inducing FASN expression by activating sterol regulatory element binding protein 1 (SREBP1) in breast cancer cells [85].

### 3.5. LDs and Hepatocarcinoma

Recently, Beradi et al. showed that Bcl2/E1B19K-interacting protein 3 (BNIP3) promoted LD turnover, inhibiting hepatocellular carcinoma (HCC) growth by limiting the cellular pool of available PLs for cellular membranes. However, this BNIP3-dependent tumor suppression mechanism may be relevant only in the liver, where lipid accumulation promotes both nonalcoholic fatty liver disease (NAFLD) and nonalcoholic steatohepatitis (NASH), which are precursor diseases to HCC [87]. 

Some studies have observed other slightly more specific functions performed by LDs. Although these studies have not been conducted on cancer models, they deserve attention. One study on a healthy liver model revealed the presence of LDs inside the nucleus. These LDs differ from cytoplasmic LDs due to their lipid composition, suggesting that lipid metabolism is modulated by LDs from inside the nucleus in order to provide the lipid mediators necessary for gene induction [88]. Moreover, it seems that cytoplasmic LDs can directly affect the activity of certain transcription factors. For example, the NFAT5 factor (nuclear factor activated T cells) appear to be associated with the Fsp27 protein, resulting in its sequestration in LDs [89]. The same year, another team showed that LDs could sequester histones, preventing their accumulation in the nucleus [90]. LDs also seem to play a major role in the process of virus maturation since viral proteins are capable of associating with LDs to initiate viral DNA replication and particle association [91].

### 3.6. LDs and Colorectal Cancer

Studies focused on CRC have already demonstrated that LDs could be the signature of a population of stem cells [92]. The presence of LDs could also make it possible to identify CRC stem cell populations presenting the CD133 marker, thus helping to predict recurrence [92]. LDs could promote proliferation by reducing the FOXO3/sirtuin6 pathway after stimulation of the epidermal growth factor receptor (EGFR) [93]. They have also been identified as the site of localization of the COX-2 enzyme and production of prostaglandin E2 (PGE2) [94]. These studies therefore suggest that these LDs are involved in the progression of CRC. However, doubts persist about their involvement in the survival of cancer cells. Indeed, some studies suggest that their accumulation characterizes cells undergoing apoptosis, whereas others have found a protective effect in cancer cells subjected to nutrient stress [83,95]. Added to these contradictions is the fact that no study so far has been interested in the potential relationship between resistance to chemotherapy and the production of LDs. This is why we recently focused on the function of LDs in CRC chemoresistance. We wanted to specify the role of PC metabolism in the production of these organelles, in particular through the LPCAT2 isoform, whose involvement in CRC we know little or nothing about. Our results showed that, among the PC production enzymes, isoform 2 of the enzyme LPCAT seemed to play a major role in the resistance phenotype, and its function seemed be exerted through the accumulation of LDs [96,97]. As mentioned above, LDs have only recently been of serious interest in the phenomena of carcinogenesis. They have been observed on many occasions, particularly in CRC, where they seem, among other things, to help cancer cells maintain their rate of proliferation and to protect them from nutritive stress caused by phenomena such as a lack of vascularization at the heart of the tumor. Another study on CRC found that LDs were involved in the proliferation of CRC cells. In this context, the accumulation of LDs is associated with a decrease in the expression of the transcription factor FOXO3 (Forkhead box protein O3) and of the deacetylase silencing information regulator 6 (SIRT6 or sirtuin6), mediated by the activation of the EGFR [93]. Similarly, another study showed that the quantity of LDs analyzed by Raman spectroscopy could be correlated with cell resistance to an EGFR inhibitor: erlotinib [98].

In cancer cells including CRC cells, lipids are typically stored in LDs. Our work based on both LPCAT2-encoding gene silencing and overexpression as well as selective inhibition of LPCAT2 enzymatic activity with TSI-01 uncovered the importance of LPCAT2 in LD accumulation in chemotherapy-resistant CRC cells [96]. Using several murine and human CRC cell models, we demonstrated that LPCAT2-mediated accumulation of LDs contributes to CRC cell resistance to several chemotherapies (e.g., 5-FU, oxaliplatine, FOX) without impacting cell growth ability, evidencing the key role of these organelles in the protection of tumor cells against intrinsic and extrinsic cell death. In vivo experiments in CT26 tumor-bearing mice confirmed that LPCAT2 overexpression favors tumor progression and resistance to FOX. LPCAT2-mediated LD accumulation impairs endoplasmic reticulum stress responses as well as caspase cascade activation in LPCAT2 overexpressing CRC cells. Interestingly, it is also associated with a reduction in immunogenic cell death and CD8^+^ T cell infiltration in CT26 syngeneic tumors engrafted in BALB/c mice and metastatic tumors of CRC patients. 

Although we demonstrated that LDs in CRC cells can modulate the function of T cells, Gabrilovich’s team showed that LDs containing electrophilic oxidatively truncated lipids in tumor-associated dendritic cells (DCs) also have this ability [99]. Tumor-associated DCs have impaired ability to cross-present antigens, thus preventing efficient antitumor-immune responses. Gabrilovitch and colleagues demonstrated that this is mechanistically associated with the accumulation in DCs of LDs specifically enriched with electrophilic oxidatively truncated lipids which bind to the major stress-induced peptide chaperone heat shock protein 70 (HSP70) and thus cause the accumulation of peptide–MHC-I complexes in late endosomes/lysosomes and prevent their translocation to the cell surface. As a result, tumor-associated DCs fail to stimulate adequate tumor-specific CD8^+^ T cell-mediated responses. Both our study and those of Gabrilovich highlight an LD-mediated mechanism of CD8^+^ T cell response regulation and suggest potential therapeutic avenues, including the targeting of LPCAT2-mediated LD accumulation, as a druggable mechanism to restore CRC cell sensitivity.

In addition, LDs have been found to be a source of eicosanoid production. This observation was first made in macrophages, eosinophils, and basophils [100]. Eicosanoid production results from the localization of enzymes involved in the synthesis of eicosanoids such as PLA2 and the extracellular signal-regulated kinase 1 and 2 (ERK 1 and 2) associated with it, as well as cyclo-oxygenase (COX-2), 5-lipoxygenase (5-LOX), 15-LOX, and FLAP (5-lipoxygenase–activating protein). The same observation was subsequently made in a cancer model. A study focusing on CRC showed that LDs are reservoirs of arachidonic acid, making it possible to group together substrates and enzymes such as COX-2 in the same organelle, creating a pro-inflammatory environment (Figure 2) [101].

### 3.7. LDs as Targets to Reverse Chemoresistance

LDs have essentially been described as biomarkers of cancer progression; however, recent studies suggest that an alteration of the latter would make it possible to counteract the phenomena of chemoresistance. Indeed, it has been shown in breast cancer cells, that an alteration of LDs biogenesis through a disruption of diacylglycerol acyltransferase 2 (DGAT2) could sensitize cancer cells to radiation [102]. Another therapeutic approach targeting LDs could use photodynamic therapy where BODIPY-based photosensitizers, BODISeI, with improved single oxygen yield and intersystem crossing, target LDs in cancer cells [103,104,105]. Another method could consist of near-infrared AIE nanoparticles (TTI), which are self-assembled nanoparticles with extremely high-efficient singlet oxygen generation efficiency, to target LDs in HCC cells [103].

## 4. Conclusions

For many decades, lipid metabolism has been the center of investigation, particularly in metabolic diseases and hyperlipidemia phenomena. The recent results presented in this review highlight the role of these lipids, the detailed description of their characteristics, which has been growing steadily over the last decades thanks to recent improvements in technology, and the role of small vesicles such as LDs in the phenomenon of carcinogenesis. Lipidomic analysis has been able to show that the composition of these vesicles could be of primary importance for the diagnosis of cancer progression.

Nevertheless, further studies are needed to assess the role of these vesicles in therapeutic response and to continue efforts to characterize their content and biogenesis. Better characterization appears essential to ensure that the roles attributed to these lipid structures are specific to them and not to other structures such as extracellular vesicles (EVs), which share many similarities with them. EVs constitute a pool of heterogeneous organelles with sizes ranging from 100 to 1000 nm diameter, thus corresponding by virtue of their size to nanovesicles, which are derived from cell membranes or produced from multivesicular bodies, and are secreted into the extracellular medium [106,107]. These EVs play various key roles in cancer progression. They have been shown to help shape the tumor microenvironment, promote immunosuppression and enhance the growth, survival, invasion and metastatic spread of cancer cells [108,109,110].

Until now, the majority of research efforts have been directed towards investigating specific classes of lipid vesicles, with limited interdisciplinary exploration of different types of lipid vesicles. LDs are closely linked to another important lipid vesicles: EVs, and a correlation has been reported between LDs and EV biogenesis. Improvements in isolation and characterization methods, such as single-vesicle analysis and imaging approaches, are poised to enhance research on the composition of EVs and LDs. As a result, scientists will be better equipped to identify specific markers that enable the discrimination of distinct EV and LD populations [111,112].

The current challenge is now important to better understand the respective composition of EV and LD in order to identify them as biomarkers but also to better appreciate their reciprocal physiological roles and their potential therapeutic applications.

## Data Availability

The authors declare that all data supporting the findings of this study are available within the article.
